# Fluorescent Molecular Imprinted Sensor Based on Carbon Quantum Dot for Nitrofen Detection in Water Sample

**DOI:** 10.3390/polym17060816

**Published:** 2025-03-20

**Authors:** Yuge Chen, Yongheng Zhou, Jinjie You, Zeming Zhang, Aili Sun, Hua Liu, Xizhi Shi

**Affiliations:** 1 State Key Laboratory for Quality and Safety of Agro-Products, Ningbo University, Ningbo 315211, China; 2 School of Marine Sciences, Ningbo University, Ningbo 315211, China

**Keywords:** molecularly imprinted polymer, carbon quantum dots, nitrofen, rapid detection, fluorescence sensor

## Abstract

The structure of nitrofen is stable and resistant to natural degradation, persisting in environments for extended periods. It can accumulate through the food chain, posing risks to human health. Here, we report a sensor based on carbon quantum dots (CQDs) and molecular imprinting technology (CQDs@MIPs). It not only possesses the specificity and stability of MIPs but also incorporates the environmental friendliness and signal amplification capabilities of CQDs, making it an ideal material for the specific detection of nitrofen residues in the environment. The interaction between CQDs@MIPs and nitrofen, as well as the successful removal of nitrofen, were confirmed through transmission electron microscopy (TEM) and Zeta potential analysis, which evaluated the morphology and particle size of the prepared CQDs@MIPs. After binding with nitrofen, the CQDs@MIP sensor exhibited a low detection limit (2.5 × 10^−3^ mg·L^−1^), a wide detection range (0.01–40 mg·L^−1^), a good linear relationship (R^2^ = 0.9951), and a short detection time (5 min). The CQDs@MIP sensor also demonstrated excellent stability, with the fluorescence intensity of CQDs@MIPs remaining above 90% of the initial preparation after 20 days. At the same time, Red, Green, Blue (RGB) color model extraction technology is used to fit the color of the sample under different concentrations, and the smart phone application is integrated to realize the visual detection of nitrofen. Furthermore, acceptable accuracy was achieved in real water samples (recovery rates ranging from 84.1% to 115.7%), indicating that our CQDs@MIP sensor has high analytical potential for real samples.

## 1. Introduction

Nitrofen, formally known as 2,4-dechlorophenylp-nitrophenylether, represents one of the pioneering diphenyl ether herbicides to be introduced in recent years [1]. It has demonstrated considerable application potential across both agricultural and forestry sectors [2]. Designed for weed control [3], it eliminates annual grasses and broadleaf weeds in crops like cotton, soybeans, and tea plantations [4]. Barnyard grass and goosegrass are efficiently controlled, exhibiting strong herbicidal performance, long duration, and high selectivity [5]. Paraquat’s molecular structure is stable, with two benzene rings, making its degradation difficult in nature [6]. Therefore, subsequent to its application, nitrofen can endure in farmland, surface water bodies, and groundwater for prolonged durations, gradually building up through the food chain and presenting potential dangers to human health [7]. People who are exposed to excessive amounts of nitrofen may experience methemoglobinemia, hemolytic anemia, and jaundice. Furthermore, this pesticide is highly irritating to the skin and eyes, and direct contact can lead to skin burns, vomiting, incontinence, hypotension, and can potentially induce methemoglobinemia and shock, posing a severe threat to human health. Traditional analytical methods such as high-performance liquid chromatography (HPLC) [8] and gas chromatography (GC) [9] have predominantly been used for the detection of nitrofen. However, these methods have various limitations, such as complex experimental instruments, high operational requirements, and expensive maintenance costs. Moreover, the analysis result is complicated by the GC or HPLC, as matrix effects from environmental samples can cause significant errors in the real-time monitoring of nitrofen. Therefore, developing a rapid, real-time, and accurate detection method for nitrofen is of significant importance for ensuring food safety and environmental monitoring.

Molecular imprinting technology (MIT) was proposed by Polyakov in 1931 [10] and is commonly described as a method for creating molecular locks that match molecular keys [11]. Molecularly Imprinted Polymers (MIPs) are engineered to alter their structure and shape, facilitating specific and complementary binding to template molecules [12]. Compared to other recognition systems, the preparation process for MIPs is straightforward and offers unique attributes such as a predictable structure, specific recognition, and broad applicability. Their remarkable physical stability, eco-friendliness, significant robustness, and cost-effectiveness have garnered widespread interest across various fields, including purification and separation [13], chemical/biosensing, artificial antibodies, drug delivery, and catalytic degradation, positioning them as a focal point in many areas [14]. Typically, there are two primary methods for producing MIPs: one is based on covalent interactions and the other is based on non-covalent interactions between the template and functional monomers [15]. The ultimate objective of molecular imprinting is to create MIPs with an affinity and specificity that rival biological receptors [16], aiming to replace these natural entities in practical applications [17]. By combining fluorescent signals with molecular imprinting technology, the development of molecularly imprinted fluorescence sensing technology enables the selective adsorption of trace pollutants and visual detection in real samples.

Carbon quantum dots (CQDs) [18,19], as novel zero-dimensional carbonaceous nanoparticles with sizes less than 10 nanometers, exhibit spherical or quasi-spherical morphologies. Given their minute size, substantial specific surface area [20], and abundant surface functional groups [21], CQDs have demonstrated significant applications in chemical sensing, imaging, catalysis, and drug delivery, and have even been utilized in near-infrared two-photon fluorescent materials. The easily adjustable structures of CQDs allow for the enhancement of their fluorescence properties, quantum yield, water solubility, and chemical stability through the surface modification of active groups or doping with heteroatoms [22]. Based on the primary sensing mechanisms, including photo-induced electron transfer (PET) [23], fluorescence resonance energy transfer (FRET) [24], and the internal filtering effect (IFE) [25], CQDs have been extensively employed to develop highly sensitive and selective fluorescent probes for the detection of organic pesticides. By combining MIPs and CQDs, CQDs@MIPs composite materials that embody the advantages of both can be synthesized. CQDs@MIPs integrate the specificity and stability of MIPs with the sensitivity and signal amplification capabilities of CQDs, making them ideal materials for the specific detection of pesticide residues. CQDs@MIPs significantly improve the insufficient selectivity of CQDs, solving the problem of identifying target molecules in complex environments. Moreover, by coating a layer of imprinted molecules around the CQDs, the specificity of detection and the stability of the material can be further enhanced. The emergence of CQDs@MIPs provides a new method for detecting herbicide residues in water bodies, agricultural products, and fishery and livestock products.

The global issue of pesticide contamination is intensifying, prompting the rapid development of technologies for detecting pesticide residues. The integration of molecular imprinting technology with CQDs has opened up a novel avenue for the detection of pesticides. In this study, we present a molecularly imprinted fluorescent sensing platform leveraging CQDs, designed for the selective identification of the herbicide nitrofen. Firstly, we optimize the synthesis time and ligand ratio and synthesize the CQDs with a fluorescence quenching effect on nitrofen. Building on this, eco-friendly and highly stable fluorescent imprinted polymer CQDs@MIPs are effectively produced using the reverse microemulsion technique. These CQDs@MIPs exhibit specificity for nitrofen detection and can proficiently eliminate the interference caused by other structurally analogous pesticides in the environment. Under the refined detection parameters, the CQDs@MIPs sensing platform ensures the satisfaction of stringent criteria regarding accuracy, precision, and sensitivity in the rapid detection of nitrofen. The study also investigates the impact of various environmental conditions on the fluorescence response and selectivity of CQDs@MIPs, demonstrating their successful application in quantifying trace amounts of nitrofen in biological samples. This method has been validated for its efficacy in rapidly, sensitively, and accurately determining the actual concentrations of nitrofen in seawater and tap water.

## 2. Experimental

### 2.1. Materials

The herbicide, Cypermethrin, Fenuron, Methoxychlor, Linuron and Benalaxyl were all purchased from DrEhrenstorfer GmbH (Augsburg, Germany), TritonX-100 was purchased from Solarbio (Beijing, China), Methacrylic acid (MAA), (3-Aminopropyl) and triethoxysilane (APTES, 99%) and Tetraethoxysilane (TEOS, 99%) were purchased from Sigma-Aldrich (Steinheim, Germany), and citric acid, urea, cyclohexane, ethanol, acetonitrile, methanol, acetic acid, dimethyl sulfoxide (DMSO), ammonia (25 wt%) and acetone were purchased from Sinopsin Group Limited (Shanghai, China). Deionized water was also obtained (18.25 MΩ cm, ultra-pure Water Purification System, Xi’an, China).

### 2.2. Instruments

The fluorescence intensity was recorded using the Fluorescence Spectrophotometer F-4600 (Hitachi, Tokyo, Japan). The morphology of the MIP-CQDs, NIP-CQDs, and CQDs was scanned using a transmission electron microscope (TEM) from Hitachi (H-7650, Japan). The surface functional moieties of the CQDs were evaluated by the FTIR Spectrum Nicolet 6700 (Thermo, Waltham, MA, USA). The UV-vis absorption spectra of the CQDs in an aqueous medium were recorded by UV-3300 (Mapada, Shanghai, China). A pure water instrument from Ningbo Xinzhi Biological Company (UPT-11-10T, Ningbo, China) was used to prepare the deionized water (18.25 MΩ cm).

### 2.3. Synthesis of CQDs

The preparation of the CQDs involved the use of an improved one-pot hydrothermal synthesis technique. At the initial stage of the experiment, 2.05 g of citric acid and 1 g of urea were precisely weighed and then dissolved in 15 mL of ultrapure water to ensure the purity and efficiency of the reaction. After 5 min of ultrasonic treatment, the homogeneous solution was transferred into a 20 mL Polytetrafluoroethylene vessel, and then the sealed reactor was placed in an autoclave at 200 °C, where it was heated continuously for 6 h. After the reaction, the high-pressure reactor was cooled to room temperature naturally, and a dark green liquid was synthesized. After freezing, the dark green powder obtained by vacuum drying was the CQDs, which were stored in the refrigerator at 4 °C until use.

### 2.4. Synthesis of CQDs@MIPs and NIPs

**Scheme 1:**
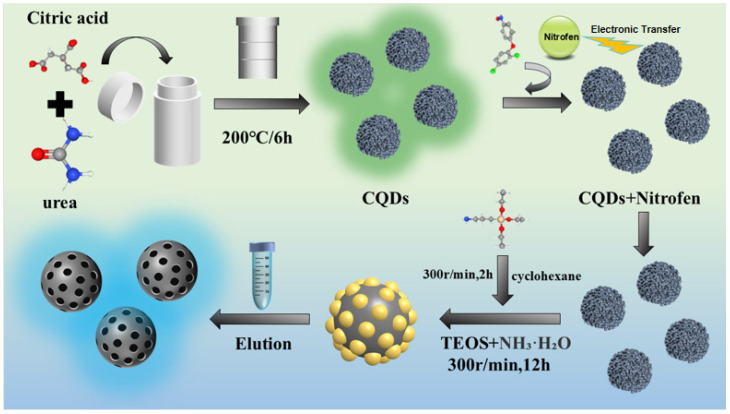
The synthetic routes of CQDs@MIPs/NIPs.

CQDs@MIPs for the specific recognition of nitrofen were prepared via an optimized reversed-phase microemulsion method. In a clean 50 mL flask, 1.8 mL of Triton X-100, 1.8 mL of ethanol, and 8 mL of cyclohexane were mixed, and this was stirred at 300 r/min for 15 min. Then, 40 μL of CQDs (110 mg/mL), 50 μL of TEOS, and 100 μL of ammonia were added successively and stirred for 2 h. Meanwhile, 1340 μL of pyrite ether (5 mg/mL), 12.8 μL of APTES, and 8 μL of MAA were pre-polymerized for 2 h and then added to a round-bottom flask and stirred at room temperature for 12 h. After synthesis, 10 mL of acetone was added to disrupt the microemulsion. The solution was transferred to a 50 mL centrifuge tube, allowed to stand until the particles started to settle, and centrifuged at 8000 g for 10 min to remove the supernatant. Next, 5 mL of ethanol was added to the precipitate, sonicated for 5 min, shaken for 45 min and centrifuged at 9000 g for 20 min. The supernatant was then sonicated to remove unreacted TEOS and APTES. Then, the precipitate was washed twice with methanol–acetic acid (9:1, *v*/*v*) and acetonitrile–ethanol (8:2, *v*/*v*) eluents to remove herbicide template molecules. Finally, the product was dried at room temperature and dissolved in ethanol. For CQDs@NIP synthesis, the herbicidal ether was replaced with an equal volume of ethanol and the above steps were followed. The specific preparation route of the CQDs@MIP sensor is listed in Scheme 1.

### 2.5. Fluorescence Determination for Nitrofen

A fluorescence spectrophotometer equipped with a 1 cm × 1 cm cuvette was employed for fluorescence detection. In the cuvette, 980 μL of ethanol was mixed with 10 μL of the CQDs@MIPs/NIPs solution (5 mg/mL). The resulting fluorescence value was recorded as F_0_. Subsequently, 10 μL of the herbicide solution was added. After a mixing reaction (5 min), the fluorescence value was recorded as F. The fluorescence values of the samples were measured using an F-4600 Hitachi fluorescence spectrophotometer. For each test, the detection steps remained consistent: the excitation wavelength was set at 335 nm, the scanning wavelength range was 370–520 nm, and the tube voltage was 700 eV.

### 2.6. Determination of Nitrofen in Water Samples

In order to minimize the influence of sample matrix, the water sample was filtered using a 0.22 μm filter membrane before detection. The seawater samples were collected from Beilun Ningbo. Then, the tap water and pure water were collected from the laboratory. Subsequently, the nitrofen-spiked samples were introduced into the water sample to determine the recovery and RSD of nitrofen. At the same time, the study established the relationship between the F_0_/F value and different concentrations of nitrofen (0.01 mg/L, 1 mg/L, 5 mg/L, 10 mg/L, 25 mg/L, 40 mg/L) and plotted a standard curve. By calculating the recovery rate and RSD, the accuracy and precision of using this method to detect seawater samples were evaluated.

## 3. Results and Discussion

### 3.1. Synthetic System Optimization of CQDs and CQDs@MIPs

The studies indicate that n-π* transition and π-π* transition have a significant impact on the fluorescence intensity. The citric acid and urea, which can provide abundant carboxyl and amino groups in the nucleation process of CQDs, promote the formation of C=O double bonds on the surface of CQDs. Thus, the ratio of citric acid to urea was systematically optimized. In this research, different ratio (3:1, 2:1, 1:1, 1:2 and 1:3) combinations were set to scan their fluorescence intensity. The findings demonstrate that the fluorescence characteristics of CQDs fabricated at diverse proportions display distinct variations, as illustrated in Figure S1A. When the molar ratio of citric acid to urea is 1:1, the F_0_ value is significantly higher than that at other ratios (reaches 5708). This indicates that CQDs prepared at this ratio exhibit the best fluorescence properties.

The high-temperature carbonization time is crucial for ensuring the quantum yield and fluorescence stability required for CQDs synthesis. The parameter was precisely regulated to determine the optimal high-temperature carbonization time. As Figure S1B shows, the fluorescence intensity of CQDs varies significantly with different carbonization times. At the beginning of the carbonization process, the fluorescence intensity of CQDs exhibits a steady increase, indicating the gradual formation of the carbon core and CQD structures. However, if the carbonization time is extended, the carbon core will accumulate, which will disrupt the stable structure of CQDs and lead to fluorescence quenching. When the carbonization time reaches at 4 h, the formation of the carbon core and structure stability reach an ideal equilibrium. This enables the fluorescence intensity and fluorescence performance of CQDs to reach the optimal state, which reveals that the optimal carbonization time is 4 h.

The ratio of template molecules to functional monomers is a key factor influencing the selectivity of CQDs@MIPs. During the synthesis of imprinted molecules, the -NO_2_ (derived from nitrofen), the -NH_2_ (derived from APTES), and the -COOH (derived from MAA) can interact via non-covalent bonds (such as hydrogen bonds) to form specific imprinted recognition sites. The ratio of APTES to MAA was fixed at 1:1 and then the ratio of APTES to the nitrofen was optimized, with ratios set at 2:1, 4:1, 6:1, 8:1, and 10:1. As depicted in Figure S1C, when the molar ratio of APTES to APTES is 6:1, the F_0_/F value of CQDs@MIPs reaches 1.969, significantly higher than the 1.561 of CQDs@NIPs. Meanwhile, the imprinting factor (IF) is 1.727, much higher than that for other ratios. These results indicate that when the ratio of APTES to nitrofen reaches 6:1, the excited CQDs@MIPs transfer electrons or energy to nitrofen, reducing its fluorescence emission.

### 3.2. Characterization of CQDs and CQDs@MIPs

The morphology of the obtained CQDs and CQDs@MIPs was investigated using the TEM and FTIR spectrum. According to the results of the electron microscopy (Figure S2), the CQDs were spherical and uniformly dispersed, with graphite fringes with a lattice spacing of about 0.21 nm being visible (Figure S2A). A particle size distribution histogram was plotted (Figure S3A) to analyze the size distribution of CQDs, revealing an average diameter of approximately 3.94 nm. As shown in Figure S2B,C, CQDs@MIPs/NIPs display regular spherule morphologies and are uniformly dispersed. These composites possess a distinct shell–core architecture, with the CQDs being centered while their outer regions are coated with SiO_2_ shells. This shell–core architecture serves as clear evidence of the successful synthesis of CQDs@MIPs/NIPs. The CQDs@NIPs appear dimmer than CQDs@MIPs, which is due to the fact that template molecules were eluted from CQDs@MIPs, leaving imprinted cavity structures on the CQDs@MIPs. The results indicate that the surface charge of CQDs is −12.3 mV, suggesting that CQDs are electronegative, with the negative charge originating from the abundant carboxyl groups provided by citric acid. The CQDs@MIPs/NIPs have positive surface charges (CQDs@MIPs: 3.4 mV, CQDs@NIPs: 2.3 mV) (Figure S3B), which is due to the positively charged groups, such as amino groups, coming from the SiO_2_ core–shell structure formed by cross-linker and functional monomers in molecularly imprinted polymers. To further verify the successful grafting of key groups onto the molecular imprinting layer, the surface functional groups of CQDs@MIPs/NIPs were analyzed by FT-IR, with the results shown in Figure S4. The vibration peaks near 1510 and 3100 cm^−1^ originate from the C=O double bond vibration on the surface of the carbon quantum dots (Figure S4A). However, in the infrared spectra of CQDs@MIPs and CQDs@NIPs, the absorption peaks at these two locations disappear. The stretching vibration absorption peak of Si-O-Si appears at 1061 cm^−1^ (Figure S4B), and the FT-IR spectra of CQDs@MIPs/NIPs are largely consistent, indicating the successful formation of the SiO_2_ core–shell structure.

### 3.3. Optimization of CQDs@MIPs Detection Conditions

Sensors have important applications in numerous fields such as environmental monitoring and food safety. However, detection sensitivity and selectivity are the key factors restricting their widespread application and further development. In order to enhance the sensitivity and selectivity of the constructed sensor, it was necessary to optimize the conditions of the detection system to improve its accuracy and reliability, allowing for more precise measurements and better discrimination between different analytes. The optimization of these critical aspects was essential to achieve a sensor that could not only detect with high sensitivity, but also selectively identify specific compounds in complex mixtures.

**Figure 1:**
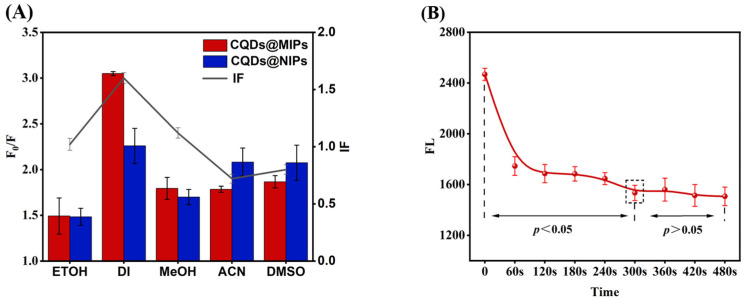
The effect of solvent (**A**) and response time (**B**) on CQDs@MIPs/NIPs.

The medium of the detection system can impact the formation of hydrogen bonds and other non-covalent interactions between the template and the functional monomer, thereby affecting the fluorescence quenching efficiency. In this study, we investigated the influence of solvents in the detection system on the fluorescence quenching efficiency. As shown in Figure 1A, in a pure water environment, the F_0_/F value of CQDs@MIPs reaches its maximum of 3.04. Compared with other solution systems, in pure water, nitrofen has a stronger fluorescence quenching effect on CQDs@MIPs than on CQDs@NIPs. This is likely due to the swelling effect of water, which exposes more recognition sites on the surface of CQDs@MIPs, allowing more nitrofen molecules to bind in a short time. Considering these findings and practical application requirements, we chose pure water as the solvent for subsequent experiments.

Generally, CQDs@MIPs need to interact with the template molecule for a certain period of time to reach a reaction equilibrium state. In this study, an investigation was conducted on the optimal response time corresponding to the fluorescence quenching equilibrium between nitrofen and CQDs@MIPs. During the experiment, 50 mg/L of nitrofen was added to the detection system, and the fluorescence intensity changes were monitored. As shown in Figure 1B, the fluorescence intensity of the system decreased significantly with the addition of nitrofen. With an increase in the reaction time, the change in the fluorescence intensity gradually leveled off. The fluorescence intensity essentially ceased to change, indicating that a dynamic adsorption equilibrium had been successfully established between nitrofen and the CQDs@MIPs when the reaction time was 5 min. To ensure that subsequent experiments could be carried out under stable and efficient conditions, the incubation time for subsequent experiments was set to 5 min.

**Figure 2:**
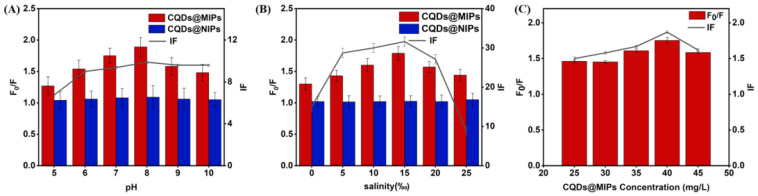
The effect of pH (**A**), salinity (**B**) and the concentration of CQDs@MIPs (**C**) on CQDs@MIPs/NIPs.

The concentration of the sensor, the acid–alkaline environment and the ion concentration significantly affect the linear range, sensitivity, and accuracy of sensor detection. As shown in Figure 2A, with an increase in pH, the fluorescence quenching efficiency (*Ksv*) of CQDs@MIPs firstly increases and then decreases. When the pH is 8.0, *Ksv* reaches its maximum, and the IF is 1.7. These results indicate that CQDs@MIPs have an excellent ability to recognize nitrofen at pH 8. Perhaps this is due to the strong acid or strong alkaline environment destroying the imprinted silicon layer, resulting in the destruction of the identification point on the polymer surface. The impact of the ion concentration on the quenching ability of CQDs@MIPs was investigated. As depicted in Figure 2B, with the increase in the surface ion concentration of CQDs@MIPs, the interaction between CQDs@MIPs and nitrofen becomes more prominent. When the ion concentration is maintained at 15‰, CQDs@MIPs and nitrofen have the highest response and the IF reaches 2.81. Figure 2C indicates that within the concentration range of 25.0–45.0 mg/L, the fluorescence quenching efficiency of CQDs@MIPs increases with the increase in the CQDs@MIPs concentration, and F_0_/F increases and reaches its maximum when the CQDs@MIPs concentration is 40 mg/L. This may be due to the excessively high concentration of CQDs@MIPs resulting in mutual aggregation and the reduced exposure of adsorption sites, thus decreasing the fluorescence quenching efficiency.

### 3.4. Performance Determination of CQDs@MIPs

The selectivity and anti-interference capacities of a sensor are key to evaluating its performance and deciding its practicality. In this study, pesticides like cypermethrin, methoxy, linuron, benalaxyl, 2,4-D, dicamba, and acetochlor, all structurally analogous to nitrofen and at twice its concentration, were chosen to assess the selectivity of CQDs@MIPs (structures in Figure S5).

**Figure 3:**
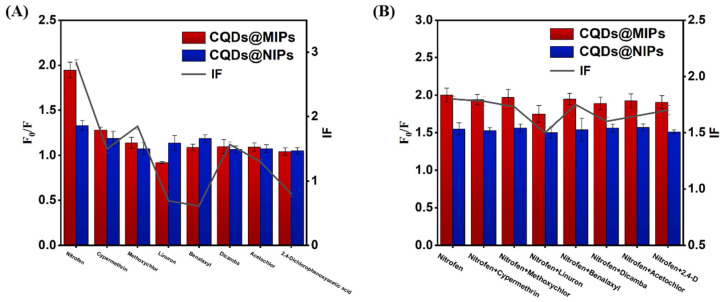
The selectivity (**A**) and competitiveness (**B**) of the CQDs@MIP sensor and CQDs@MIP sensor to nitrofen and other interferents.

As shown in Figure 3A, at 20 mg/L nitrofen, the initial fluorescence intensity (IF) of CQDs@MIPs reaches 1.947, much higher than that for other analogues. The fluorescence quenching of analogues is far lower than that of nitrofen, while CQDs@NIPs show similar fluorescence responses to all substances. This indicates that CQDs@MIPs can specifically identify nitrofen among structurally similar substances due to the specific binding sites on its surface, which are hard for other analogues to bind to.

Then, the anti-interference ability of CQDs@MIPs was assessed. As shown in Figure 3B, when nitrofen is introduced, a significant change in the F_0_/F ratio of CQDs@MIPs is observed. However, when analogues at twice the concentration of nitrofen are added to the CQDs@MIP solution, the F_0_/F ratio shows no significant change. This clearly demonstrates that, under such concentration conditions, the binding affinity between nitrofen and the CQDs@MIPs remains strong, even in the presence of interfering structural analogues. Consequently, CQDs@MIPs exhibit strong anti-interference properties and can reliably detect nitrofen in complex environments.

**Figure 4:**
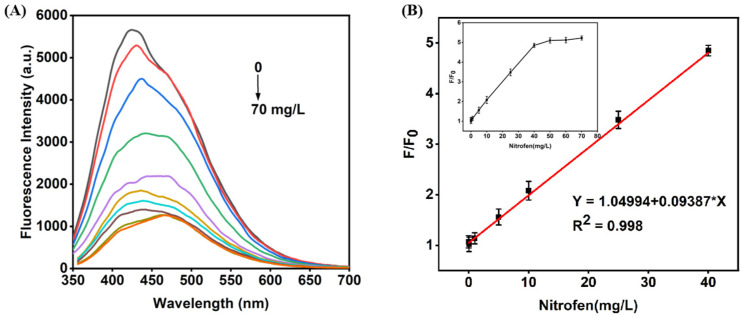
CQDs@MIP fluorescence spectrum (**A**) and its standard (**B**) at different nitrofen concentrations.

Under the optimized detection system, the reaction observed when adding different concentrations of nitrofen was studied. As shown in Figure 4, nitrofen was gradually added to the CQDs@MIP solution. When the concentration of nitrophenol is within the range of 0–70 mg/L CQDs@MIPs, it gradually decreases until saturation. Subsequently, the Stern Volmer equation was used to fit the standard curve. Regarding the concentration of 0–40 mg/L nitrofen, F_0_/F = 1.050 + 0.094x is given, and the determination coefficient (R^2^) is 0.998 (Figure 4B). This indicates a strong linear correlation between the nitrate concentration and the fluorescence quenching reaction. This highlights its significance in quantitative analysis.

**Figure 5:**
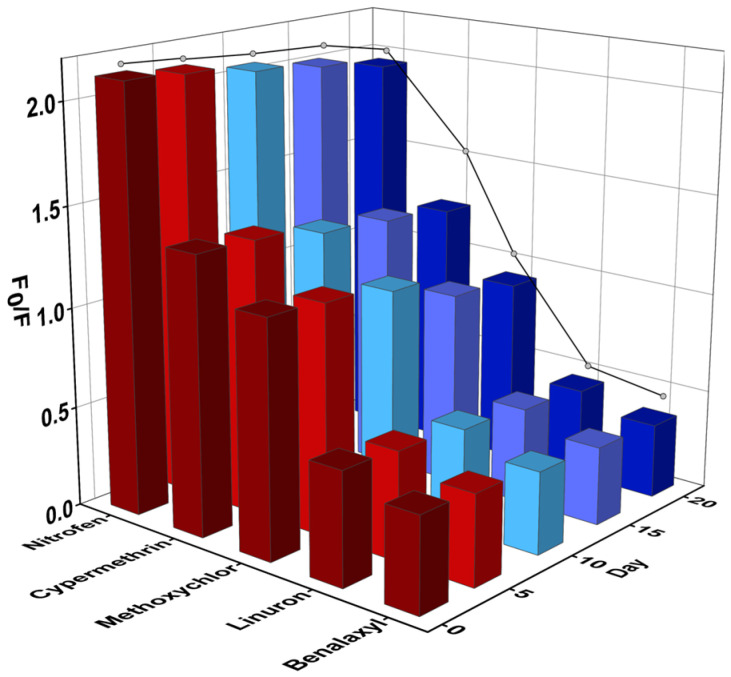
Effect of time on the fluorescence intensity and the ability to selectively recognize nitrofen.

To investigate the stability, the fluorescence changes in the CQDs@MIPs were monitored over a period of 20 days, as illustrated in Figure 5; after this period, it was observed that the fluorescence intensity of the CQDs@MIPs retained more than 90% of its initial value at the time of preparation. This finding suggests that the CQDs@MIPs prepared in this study possess excellent stability, which is essential for ensuring the reproducibility of the experimental results. Moreover, the excellent stability also indicates that CQDs@MIPs are a promising candidate for long-term and continuous monitoring applications, providing reliable data over extended periods without the significant degradation of performance. The reproducibility of the results obtained using CQDs@MIPs further validates their potential utility in various analytical and sensing applications, where consistent and accurate measurements are essential requirements.

From the above analysis, it can be inferred that the fluorescence quenching of CQDs@MIPs might be attributed to the electron or energy transfer process that occurs during its interaction with nitrofen. This process leads to the transfer of electrons or energy from the excited CQDs@MIPs to nitrofen, thereby reducing its fluorescence emission capabilities. Moreover, due to the unique molecular imprinted structure of CQDs@MIPs, the binding of the recognition sites on their surface to nitrofen affects the internal electron cloud distribution and energy level structure. As a consequence, the fluorescence emission process is inhibited, resulting in fluorescence quenching.

### 3.5. Real Samples Analysis

The practical application of the CQDs@MIP sensor is demonstrated through the analysis of real samples, such as the seawater, the tap water, and the purified water. The practical application of the CQDs@MIP sensor was evaluated using the relative standard deviation (RSD) and recovery rates. As shown in Table 1, the results of the standard addition recovery experiments for real samples indicate that the recovery rate of nitrofen in the seawater samples using the CQDs@MIP sensor was between 84.1 and 115.7%, with an RSD below 3.1%. The recovery rate of nitrofen in the tap water samples was between 86.6 and 103.7%, with an RSD below 4.7%. The recovery rate of nitrofen in the purified water was between 96.5% and 103.3%, with an RSD below 6.7%. The detection limit, calculated using LOD = 3σ/S (where σ is the relative standard deviation of ten consecutive blank sample measurements, and S is the slope of the standard curve), reaches 1.49 mg/L, enabling the detection of nitrofen at very low concentrations, providing strong technical support for environmental monitoring and food safety. When analyzing real samples, the sensor does not require complex sample pretreatment steps, reducing the waiting time. These results not only verify the effectiveness of the sensor under laboratory conditions, but also demonstrate its potential for long-term monitoring applications in the field of rapid on-site detection.

Food safety is a global issue, and the harmful substances in food pose a serious threat to human health. Consequently, the detection of food contaminants is crucial. The traditional detection methods have many limitations related to their use of expensive equipment, their complex operations, and their time-consuming and labor-intensive nature. As a result, it is difficult to meet the needs of rapid and accurate detection. Metal–organic frameworks (MOFs), quantum dots (QDs), and CQDs have demonstrated remarkable potential in the field of food contaminant detection due to their distinctive properties. MOFs possess high porosity, a tunable composition and structure, and excellent stability, which render them efficient platforms for constructing sensors. CQDs exhibit outstanding photoelectric properties, biocompatibility, and a structure that is amenable to being functionalized and modified. When integrated with MIT, they can enhance the selectivity and sensitivity of sensors. Ongoing research has achieved results regarding the detection of various pollutants with good accuracy and precision.

The CQDs@MIP sensor exhibits specificity for detecting nitrofen and can effectively mitigate the interference caused by structurally similar pesticides in the environment. The papers presented in Table 2 focus on the domain of food safety, demonstrating the application results of related materials in detecting nitrofen. In comparison with the results reported in the literature, the CQDs@MIPs present a broader linear range and excellent sensitivity. These advantageous features can be attributed to the remarkable photoelectric properties of CQDs and the specific recognition ability of MIPs. These characteristics make CQDs@MIPs an ideal choice for detecting pesticide nitrofen residues in water environments.

### 3.6. Performance Evaluation of Deep Learning in RGB Analysis

**Figure 6:**
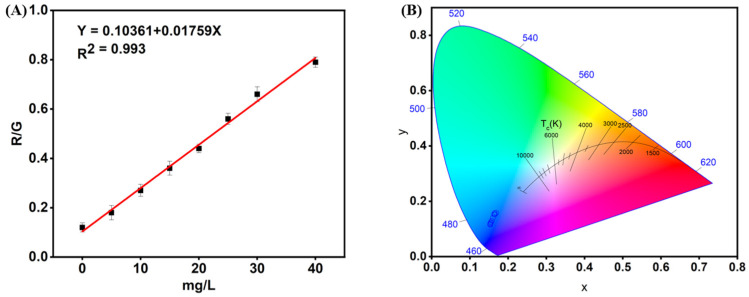
Regression analysis of CIE and linear analysis.

The RGB (red, green, blue) color model is the most common method used for representing colors. By mixing the three primary colors of red, green, and blue in different proportions, nearly all visible colors can be produced. With the continuous development of image processing technology, the accurate extraction and analysis of RGB is of great importance in many fields such as computer vision, art design, and industrial inspection. To solve the problem of the portable detection of nitrofen in the environment, this study used a smartphone-based fluorescent sensor to achieve the visual detection of nitrofen. As shown in Figure 6B, the fluorescence color of the sensor gradually changes with the concentration of nitrofen, consistent with the color trend in the CIE chromaticity coordinate diagram. A smartphone is used as a colorimeter to capture fluorescence images and obtain RGB values. As shown in Figure 6A, the linear relationship equation between the R/G value of the fluorescence image and the herbicide concentration (0–40 mg/L) is y = 0.01759x + 0.10361, with R^2^ = 0.993. Meanwhile, the calculated LOD value is 3 g/L, similar to the result of the fluorescence analysis. These results indicate that the sensor combined with smartphone software has the ability to perform highly sensitive visual analysis.

## 4. Conclusions

In this work, we designed and created an MIP-based CQD sensor for detecting nitrofen in real seawater and tap water samples. The CQDs were synthesized to specifically quench the fluorescence of nitrofen. CQDs@MIP sensors were created using an optimized reversed-phase microemulsion method to enhance stability and cost-effectiveness. The detection conditions were set as follows: a 40 mg/L concentration, a 5 min time, and pure water medium; this resulted in an improved imprinting performance and the specific recognition of nitrofen. The results indicated that the CQDs@MIPs had enough specificity for the detection of nitrofen and could effectively eliminate the interference caused by other pesticides with similar structures in the environment. Under the optimized system, we can use the sensor for nitrofen detection within the concentrations of nitrofen (0–50 mg/L), which are associated with a limit of detection (LOD) of 2.5 × 10^−3^ mg/L; the standard curve fitting showed that the fluorescence intensity of CQDs@MIPs exhibited a good linear relationship with nitrofen (R^2^ = 0.9951). The CQDs@MIP sensor was used to detect actual seawater and tap water. The recovery rates were 84.1–115.7%, and the RSD was less than 6.7%. Through the validation of actual samples, a high specificity and sensitivity were achieved to ensure the accuracy of the results. Our scheme offers a cost-effective platform for detecting nitrofen, using eco-friendly carbon quantum dots instead of metal ones, which reduces the environmental threats posed by heavy metals and provides a reliable method for detecting other pollutants.

## Figures and Tables

**Table 1:** The practical application of the CQDs@MIP sensor.

Sample	Spiked Concentration (mg/L)	Detection Concentration (mg/L)	Recovery Rate (%)	RSD
Seawater	1.00	1.08	108.1	1.1%
	10.00	8.41	84.1	3.1%
	50.00	57.88	115.7	1.9%
Tap water	1.00	0.86	86.6	3.1%
	10.00	10.15	101.5	4.7%
	50.00	51.84	103.7	2.1%
Purified water	1.00	1.03	103.3	2.9%
	10.00	10.18	101.9	6.7%
	50.00	48.25	96.5	2.6%

**Table 2:** The literature reported in the detection of nitrofen in complex media.

Biosensor	Analyte	Linear Range (mg/L)	LOD (mg/L)	References
nano-BCL@MAC-ZIF-8	Nitrofen	0–22.5	0.13	[26]
3D ABW-type Z-MOF	Nitrofen	0.8~20.00	57	[27]
CRL@UIO-66/Pro	Nitrofen	/	2.93	[28]
Fe_3_O_4_@MOF	Nitrofen	0.002–0.3	6 × 10^−4^	[29]
HPLC	Nitrofen	0.005~0.1	1.56 × 10^−5^	[30]
CDs@MMIP	Nitrofen	0.04~0.25	5 × 10^−5^	[31]
CQDs@MIPs	Nitrofen	0.01~40	2.5 × 10^−3^	This work

## Data Availability

Data will be made available upon request.

## Supplementary Materials

The following supporting information can be downloaded at: https://www.mdpi.com/article/10.3390/polym17060816/s1, Figure S1: (A) Optimization of citric acid to urea ratio of CQDs, (B) The high temperature carbonization time of CQDs, (C) Effect of different APTES dosage on the fluorescence quenching effect of CQDs@MIPs; Figure S2: TEM images of (A) CQDs, (B) CQDs@MIP and (C) CQDs@NIP; Figure S3: Particle size of CQDs (A) and electric potential of CQDs, CQDs@MIPs and CQDs@NIPs (B); Figure S4: FT-IR spectra of (A) CQDs, (B) CQDs@MIP and CQDs@NIP; Figure S5: The structural formula of Nitrofen and its structural analogues.
