# Supplementary material for: Fluorescent Molecular Imprinted Sensor Based on Carbon Quantum Dot for Nitrofen Detection in Water Sample
Source: Polymers (Basel). 2025 Mar 20;17(6):816. doi: 10.3390/polym17060816 (PMC11944888; doi:10.3390/polym17060816)
Supplement: Supplementary file 1 [file polymers-17-00816-s001.zip › polymers-3492692-supplementary.pdf]

Fluorescent Molecular Imprinted Sensor Based on Carbon Quantum Dot for Nitrofen Detection in Water Sample

Yuge Chen [1,2], Yongheng Zhou [1,2], Jinjie You [2], Zeming Zhang [1,2], Aili Sun [2], Hua Liu [1,2,*] and Xizhi Shi [1,2,*]

[1] State Key Laboratory for Quality and Safety of Agro-Products, Ningbo University, Ningbo 315211, China.
[2] School of Marine Sciences, Ningbo University, Ningbo 315211, China.
* Correspondence: Author: Prof. Xizhi Shi, School of Marine Sciences, Ningbo University, 818 Fenghua Road, Ningbo 315211, P.R. China, E-mail: shixizhi@nbu.edu.cn, Phone: +86-574-87609582.

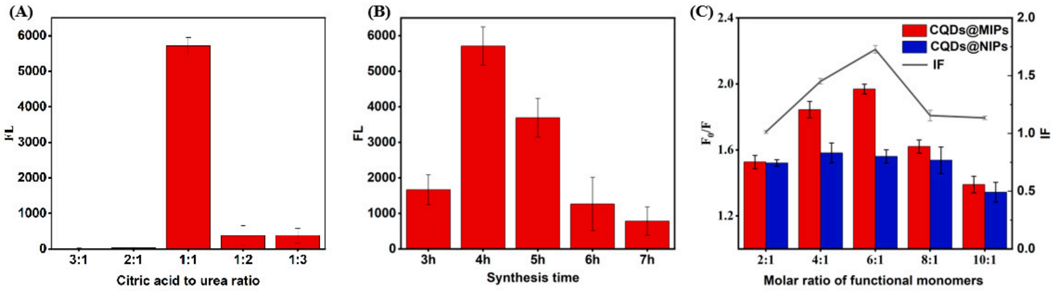

Figure S1. (A)Optimization of citric acid to urea ratio of CODs (B)The high temperature carbonization time of CODs (C)Effect of different APTES dosage on the fluorescence quenching effect of CQDs@MIPs.

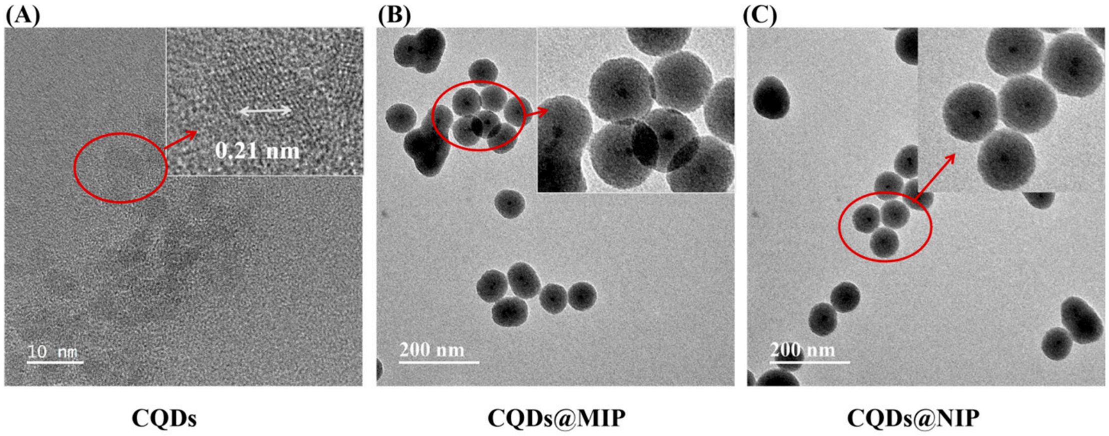

Figure S2. TEM images of (A) CQDs , (B) CQDs@MIP and (C) CQDs@NIP.

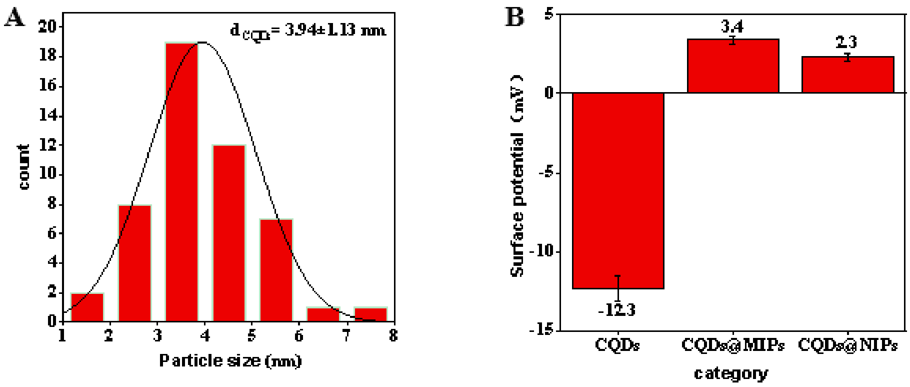

Figure S3. Particle size of CQDs(A) and electric potential of CODs,CQDs@MIPs and CQDs@NIPs(B).

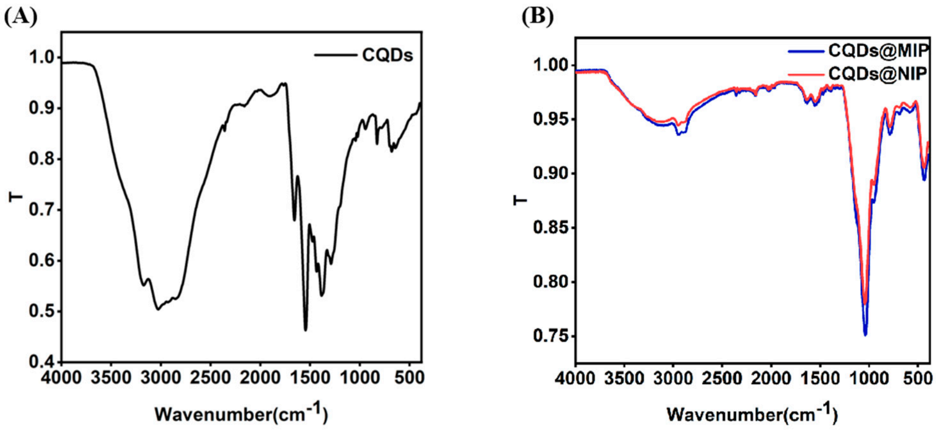

Figure S4. FT-IR spectra of (A)CQDs , (B)CODs@MIP and CODs@NIP.

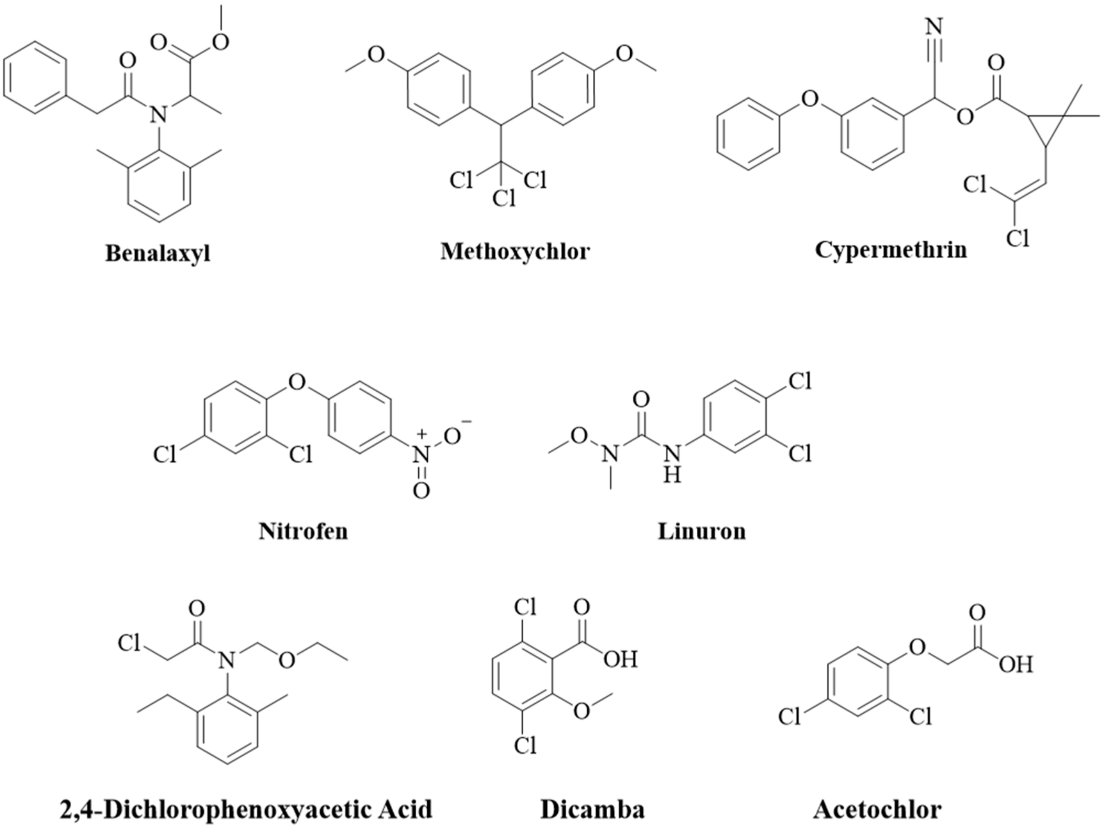

Figure S5. The structural formula of Nitrofen and its structural analogues.
